# Tablet formulation development focusing on the functional behaviour of water uptake and swelling

**DOI:** 10.1016/j.ijpx.2021.100103

**Published:** 2021-11-02

**Authors:** Jan Lenz, Jan Henrik Finke, Heike Bunjes, Arno Kwade, Michael Juhnke

**Affiliations:** a Novartis Pharma AG, Fabrikstrasse 2, CH-4056 Basel, Switzerland; b Technische Universität Braunschweig, Institut für Partikeltechnik, Volkmaroder Strasse 5, D-38104 Braunschweig, Germany; c Technische Universität Braunschweig, Zentrum für Pharmaverfahrenstechnik - PVZ, Franz-Liszt-Strasse 35a, D-38106 Braunschweig, Germany; d Technische Universität Braunschweig, Institut für Pharmazeutische Technologie und Biopharmazie, Mendelssohnstrasse 1, D-38106 Braunschweig, Germany

**Keywords:** Water uptake, Swelling, Tablet performance, Pharmaceutical polymers, Amorphous solid dispersions, Gelling, Modelling

## Abstract

The functional behaviour of tablets is strongly influenced by their manufacturing process and the choice of excipients. Water uptake and swelling are prerequisites for tablet disintegration, dispersion and hence active pharmaceutical ingredient (API) dissolution. High proportions of polymeric excipients in tablets, which are typically used as API carriers in amorphous solid dispersions (ASDs), may be challenging due to the formation of a gelling polymer network (GPN). In this study, systematic investigations into the formulation development of tablets containing polymeric and other excipients are performed by water uptake and swelling analysis. The impact of tablet composition and porosity as well as pH of the test medium are investigated. The pH affects the analysis results for Eudragit L100–55 and Eudragit EPO. HPMC and Kollidon VA64 inhibit water uptake and swelling of tablets due to the formation of a GPN. High tablet porosity, coarse particle size of the polymer and the addition of fillers and disintegrants can reduce the negative impact of a GPN on tablet performance. The application of lubricants slows down the analysed processes. Water uptake and swelling data are fitted to an empirical model obtaining four characteristic parameters to facilitate the simple quantitative assessment of varying tablet formulations and structural properties.

## Highlights

• evaluation of tablet formulations containing pharmaceutical polymers by water uptake and swelling analysis with different liquid medium
• empirical model describing water uptake and swelling of tablets with 4 characteristic parameters
• improved mechanistic understanding of processes of pore penetration and swelling through linkage of water uptake and swelling data

## Graphical abstract

**Figure:**
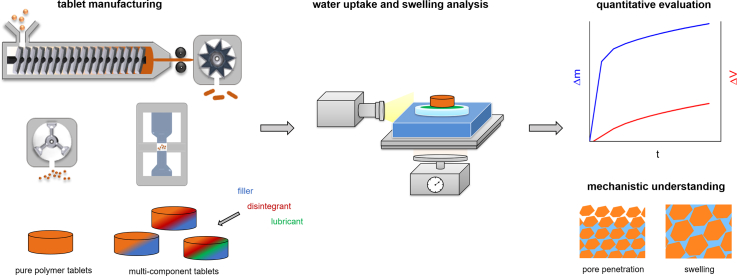

## 1 Introduction

A high number of active pharmaceutical ingredients (API) developed in the last decades are poorly water-soluble. Around 40% of the approved immediate-release oral drugs are even categorised as practically insoluble (< 0.1 mg/ml) in water (Liu, 2018; Takagi et al., 2006). These substances are classified in class II or IV according to the Biopharmaceutics Classification System, depending on their intestinal permeability (Amidon et al., 1995). A sophisticated formulation strategy for BCS class II or IV substances is crucial to increase their bioavailability. Various approaches to enhance the dissolution properties of APIs have been commonly applied, including crystal modification, particle size reduction and amorphisation (Kawabata et al., 2011). One strategy to achieve the latter is the dispersion of API molecules in an amorphous polymeric carrier, also referred to as glass solution, representing the most commonly formulated type of an amorphous solid dispersion (ASD) (Taylor and Zhang, 2016; Schittny et al., 2020). Spray drying and hot melt extrusion are the most frequently used manufacturing processes for ASDs (Janssens and Van den Mooter, 2009; Van den Mooter, 2012).

Tablets containing ASDs pose several inherent challenges regarding the tablet formulation development, manufacturing and performance. Demuth et al. pointed out four critical attributes of the materials to be compressed, namely grindability, flowability, compressibility and the disintegration of such tablets (Demuth et al., 2015). The cause that disintegration is a critical attribute is to be found in the propensity of the tablets to form a gelling polymer network (GPN) upon contact with water, affecting the tablet performance and hence API release. Some of these challenges can be overcome by the addition of suitable excipients, such as fillers, disintegrants or lubricants. High proportions of fillers and disintegrants are often required, impairing the maximum load of the polymeric carrier, respectively the maximum API dose strength (Demuth et al., 2015; DiNunzio et al., 2012; Goddeeris et al., 2008). The use of lubricants can negatively impact the water uptake and disintegration behaviour of tablets (Late et al., 2009; Bele and Derle, 2012). Thus, the development of multi-component formulations and a thorough understanding of the complex processes during application are crucial, which can be facilitated by using appropriate analytical methods.

Dissolution tests represent an in vitro method to obtain API release profiles in an aqueous dissolution medium, thus assessing performance and quality attributes of tablets in formulation development and manufacturing (Gray, 2018; Limberg and Potthast, 2013). DiNunzio et al. observed gelling and hence a reduction of the dissolution rate of tablets containing MCC as filler, 10% cross-linked sodium carboxymethyl cellulose (NaCMCXL) and at least 50% ASD based on the polymeric carrier Kollidon VA64 (DiNunzio et al., 2012).

Typically, solid dosage forms undergo a variety of subprocesses until API release, including wetting, water uptake, swelling, disintegration, dispersion and dissolution (Almoazen, 2013; Markl and Zeitler, 2017; Quodbach and Kleinebudde, 2016). Each of these subprocesses represents a characteristic of the tablet performance and thus can have an impact on the API release. Disintegration time, the time needed to break up tablets into agglomerated or primary particles in a dissolution medium, is commonly determined beside dissolution (Basha et al., 2020; Hirasawa et al., 2004; Yu et al., 2020). Xi et al. reported of strongly increasing disintegration times with increasing polymer content in tablets containing MCC, Kollidon VA64, NaCMCXL and magnesium stearate (MgSt), which were related to the formation of a GPN hindering water penetration into the tablets (Xi et al., 2020). Yu et al. investigated the impact of different disintegrants on the disintegration time of tablets. They found that tablets containing polyvinyl polypyrrolidone (PVPP) showed faster disintegration compared to carboxymethyl starch sodium and low-substituted hydroxypropyl cellulose. Furthermore, similar disintegration times were achieved with 3% and 5% polyvinyl polypyrrolidone (Yu et al., 2020). Goddeeris et al. compared the disintegration times of tablets containing ASDs based on vinylpyrrolidone vinylacetate copolymer (Kollidon VA64) and hydroxypropyl methylcellulose (HPMC). 25% PVPP was used as disintegrant and microcrystalline cellulose (MCC) was used as filler with varying content. The disintegration behaviour was found to be acceptable for formulations with Kollidon VA64 as polymeric carrier and 75% MCC. In contrast, disintegration of tablets containing HPMC was hindered due to its potential to form a gel (Goddeeris et al., 2008). However, the relevance and discrimination power of the conventional disintegration test according to USP <701> is currently a matter of debate (Kindgen et al., 2016; Markl and Zeitler, 2017; Quodbach and Kleinebudde, 2014a).

A preceding and necessary requirement for disintegration and dissolution is the proper wetting of the tablet with the dissolution medium initiating the subprocesses water uptake and swelling. The medium penetrates into the porous system of the tablet driven by capillary forces (Bala et al., 2012; Wan and Heng, 1987). Furthermore, hydrophilic polymeric excipients can absorb water eliciting a volume expansion, which is defined as swelling (Colombo et al., 1999; Schott, 1992; Schott, 2006). The swelling breaks up bonds between particles in a tablet and promotes disintegration. Thus, the exposure of surface area of primary particles in a tablet to the dissolution medium is enhanced and, correspondingly, API release is facilitated (Markl and Zeitler, 2017).

In an earlier study, the state of the art of analytical approaches was presented and a method to simultaneously quantify the increase of mass and volume of tablets upon water uptake through their front face was introduced (Lenz et al., 2021). This method provided comprehensive information about the complex interplay of both subprocesses.

Several approaches of modelling water uptake and swelling of tablets have been developed. In general, water penetration through porous media can be described by Darcy's law or the Washburn equation (Darcy, 1856; Washburn, 1921). Both models are based on the assumption of a constant tablet geometry and structure of the pores during the penetration process. In fact, swelling leads to considerable changes of the tablet geometry and structure and, thus, the applicability of a model describing the pore penetration is limited (Markl and Zeitler, 2017) (Markl and Zeitler, 2017). Ferrari et al. derived an empirical model based on the Hill equation for the fitting of tablet water uptake data. In order to discriminate the water uptake amount induced by pore penetration and swelling, they described the processes separately with two accumulated equation terms of the Hill equation (Ferrari et al., 1991). However, as the model was applied to the measurement data of water uptake of tablets only, there was no experimental discrimination of water uptake and swelling possible. Quodbach and Kleinebudde similarly employed the Hill equation for the fitting of water uptake data (Quodbach and Kleinebudde, 2014b). Korsmeyer et al. modelled the drug release from swelling, porous polymeric systems with a power law and obtained information about the kinetics and mechanisms of different formulations (Korsmeyer et al., 1983). Yassin et al. similarly described the development of the water penetration front of tablets with a power law (Yassin et al., 2015a; Yassin et al., 2015b). So et al., Kimber et al. and Siepmann et al. developed models based on Fick's second law to describe the diffusion-controlled processes of water transport and swelling, in order to gain an improved understanding of tablet disintegration (So et al., 2021) or drug release, respectively (Kimber et al., 2012; Siepmann et al., 1999). Markl et al. proposed an approach to model both water transport and swelling of tablets, which was based on Darcy's law for pore penetration and an empirical second-order kinetics derived by Schott for swelling of polymers. The model included the consideration of changes of pore size, porosity and permeability during the process. Detailed material and tablet characteristics were employed for the approach to predict water uptake and swelling. However, the empirical determination of some parameters was also necessary for the modelling approach. A very beneficial aspect of their work was the use of two separate experimental data sets, one describing water transport and another describing swelling of the same tablet sample, determined by terahertz pulsed imaging. During those measurements, only the tablet front face was in contact with water and radial expansion was restricted by the tablet holder. Thus, the data described the unidirectional axial water penetration and swelling processes (Markl et al., 2017; Schott, 1992; Yassin et al., 2015a, Yassin et al., 2015b).

Although several researchers have simultaneously investigated water uptake and swelling of tablets, none of these methods has been applied to specifically investigate tablets containing ASDs or relevant excipients, such as polymeric carriers.

The aim of this study is the quantitative evaluation of tablets of pure polymers considered for the use as polymeric API carriers in ASDs and related multi-component formulations regarding water uptake and swelling. The impact of polymer type and particle size, additional excipients, such as fillers, disintegrants and lubricants, tablet structural properties, such as porosity, and pH of the water is investigated. Water uptake is measured with a balance and the swelling of the tablet is determined with an optical imaging technique, according to an experimental set-up reported in an earlier study (Lenz et al., 2021). Furthermore, an empirical model is introduced depicting the experimental results for water uptake and swelling with four characteristic parameters.

## 2 Materials and Methods

### 2.1 Materials

Five pharmaceutical polymers, which are commonly used as carriers for the manufacturing of ASDs, hydroxypropyl methylcellulose acetate succinate (HPMCAS, AQOAT AS-LG, Shin-Etsu, Venlo, Netherlands), methacrylic acid copolymer (Eudragit L100–55, Evonik, Essen, Germany), aminoalkyl methacrylate copolymer (Eudragit EPO, Evonik, Essen, Germany), hydroxypropyl methylcellulose (HPMC, AFFINISOL HPMC HME 15 cP, DuPont, Milano, Italy) and vinylpyrrolidone vinylacetate copolymer (Kollidon VA64, BASF, Basel, Switzerland) were investigated (Baghel et al., 2016; Shah et al., 2014). Microcrystalline cellulose (MCC, Vivapur 102, JRS Pharma, Rosenberg, Germany), a hydrophilic and water-insoluble excipient, and mannitol (PEARLITOL 200 SD, Roquette, Vernier, Switzerland), a hydrophilic and water-soluble excipient, were used as fillers (Paul et al., 2018; Thoorens et al., 2014). Cross-linked sodium carboxymethyl cellulose (NaCMCXL, AcDiSol SD-711, FMC Europe NV, Brussels, Belgium) and polyvinyl polypyrrolidone (PVPP, Polyplasdone XL, Ashland, Schaffhausen, Switzerland) were employed as disintegrants. Magnesium stearate (MgSt, Magnesium Stearate EUR.PHAR., FACI, Carasco, Italy) and sodium stearyl fumarate (SSF, PRUV, JRS Pharma, Rosenberg, Germany) were used as lubricants. Throughout the study, demineralised water with a pH of 7 and 0.1 N hydrochloric acid (Titripur, Merck, Zug, Switzerland) with a pH of 1 were used as test media at a temperature of 22 ± 2 °C.

### 2.2 Polymer powder preparation

All pharmaceutical polymers were processed in a hot-melt extrusion process with a twin-screw extruder (Pharma 11, Thermo Fisher Scientific, Basel, Switzerland) with a 2 mm rod die. The process parameters were adjusted for each polymer in order to obtain non-porous extrudates. Subsequently, the extrudates were cut into pellets with a length of 3 mm using a pelletiser (VariCut, Thermo Fisher Scientific, Basel, Switzerland). A small amount of polymer extrudates was retained and used as test specimens for a complementary water uptake analysis. The pellets were milled to obtain powders for tablet manufacturing. Eudragit L100–55, Eudragit EPO and Kollidon VA64 were milled with a hammer mill (MF 10/10.2, IKA, Staufen, Germany) at 4′000 rpm, where the product passed through an inserted sieve with 500 μm mesh size (MF 0.5, IKA, Staufen, Germany). In addition, two finer grades of Kollidon VA64 powder were prepared using a rotor impact pin mill (63UPZ, Hosokawa-Alpine, Augsburg, Germany) at 12′000 and 27′500 rpm, respectively. HPMCAS and HPMC were milled with a rotor impact pin mill (100UPZ, Hosokawa-Alpine, Augsburg, Germany) at 20′000 rpm.

### 2.3 Particle size measurement

The particle size distributions of the polymer powders were determined in wet dispersed condition by laser diffraction (HELOS/KR + QUIXEL, Sympatec, Clausthal-Zellerfeld, Germany) according to Fraunhofer theory. n-heptane (n-heptane 99%, Brenntag, Basel, Switzerland) was used as dispersion medium for Eudragit EPO and White Spirit (White Spirit/Terlitol 16/18%, Brenntag, Basel, Switzerland) was used as dispersion medium for all other polymers.

### 2.4 Mercury porosimetry

A mercury intrusion porosimeter (PoreMaster 60 GT, Quantachrome/Anton Paar, Buchs, Switzerland) was employed to determine the average pore size d_50,3_ of pure pharmaceutical polymer tablets. Applied intrusion pressures between 0.03 and 147 MPa and correspondingly pore sizes between 50 and 0.01 μm were considered as the relevant range of tablet pores in the analysis.

### 2.5 Tablet manufacturing

Formulations of two or more excipients were blended with a laboratory mixer (Turbula, Willy A. Bachofen AG, Basel, Switzerland) at 34 rpm for 20 min. Throughout this study, the declared compositions of formulation components were related to their mass.

A single punch tablet press (Styl'one Evolution, Medelpharm S.A.S., Beynost, France) was used for the preparation of round flat-faced tablets with a constant tablet mass of about 500 mg. Compressibility profiles were generated for each formulation in a range between 20 and 300 MPa to obtain specific tablet porosities. Throughout all compaction processes, the compaction profile StylCamDirectCam with a compaction speed of 5 rpm was applied using 11.28 mm Euro-D punches and a paddle feed shoe.

The diameter and height of each tablet were measured by a micrometre (IP65, Mitutoyo Schweiz AG, Urdorf, Switzerland) and the mass was determined by an external balance (AT261 DeltaRange, Mettler Toledo Schweiz GmbH, Greifensee, Switzerland) prior to the measurement. These values were used to calculate the tablet volume, porosity and total pore volume, in consideration of the densities of the excipients. A relaxation period of 7 days at controlled room temperature (22 ± 2 °C) and humidity (35 ± 5%rh) was considered after tableting.

### 2.6 Water uptake of polymer extrudates

Cylindrical polymer test specimens with a length of 25 mm and a diameter between 2.2 and 3.8 mm were immersed in water with a pH of 7 and a pH of 1, respectively. After certain time steps, the mass was analysed with a balance (XP205, Mettler Toledo Schweiz GmbH, Greifensee, Switzerland). Water drops formed at the surface of the test specimens were carefully removed with a wipe prior to weighing. The normalised mass increase was calculated according to Eq. 1:

(1) $$∆m_{\mathrm{ext},\mathrm{norm}}\left(t\right)=\frac{m\left(t\right)-m\left(t=0\right)}{S\left(t=0\right)}$$

with normalised mass increase Δm_ext,norm_(t), mass of the extrudates, m(t), mass of the extrudates at *t* = 0, m(t = 0), and surface area of the extrudates at t = 0, S(t = 0).

### 2.7 Water uptake and swelling of tablets

The method for the simultaneous and time-resolved analysis of water uptake and swelling of tablets used in this study was presented earlier (Lenz et al., 2021). In brief, the experimental set-up was designed to allow water penetration through the tablets' front face. Water uptake was determined with a balance by measuring the increase of tablet mass with a data readout frequency of 7 Hz. Swelling was determined by recording 2D side view images of the tablets with a digital camera at 22 frames per second. A self-developed algorithm for the symmetry-based 3D volume reconstruction was applied to obtain volumes of the tablets from 2D images.

### 2.8 Water penetration in tablets

A complementary qualitative analysis of water penetration into the tablets was performed using 1% *w*/w methylene blue (Sigma-Aldrich, Buchs, Switzerland) to dye the water prior to water uptake and swelling measurements. After a measurement duration of 1000 s, the tablets were carefully removed from the tablet holder and symmetrically cut in axial direction. A light microscope (Axio Zoom.V16, Zeiss, Jena, Germany) was used to take images of the cut surface of the tablets.

### 2.9 Modelling of water uptake and swelling of tablets

The analytical method employed in this work provided two individual datasets from the measurement of a single tablet, separately characterising the processes of water uptake and swelling. Due to the interplay of both processes, a linked mathematical model for water uptake and swelling was derived. There were three basic considerations for the empirical model used in this study to describe these processes for tablets containing pharmaceutical polymers.

**Fig. 1:**
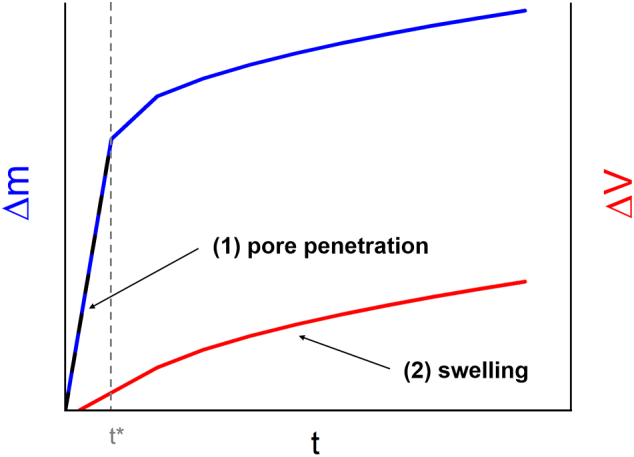
Illustration of the theoretical subdivision of water uptake and swelling data in two sections according to the dominating mechanism, which changes at the transition point t*: (section 1) pore penetration, (section 2) swelling.

1) The mechanism for water uptake was either pore penetration or swelling, or a combination of both.

2) The mechanism for swelling was either diffusion of water into the polymer or increase of the pore volume, or a combination of both.

3) The dominating mechanism for water uptake was pore penetration initially (section 1), which changed to swelling at a transition point (section 2). The two sections and the transition point are illustrated in Fig. 1.

The empirical model consisted of three parts, expressed in separate mathematical equations for each part.

Firstly, a simplified expression for the initial pore penetration was employed. The flow of a liquid through a porous medium is frequently described with Darcy's law or Washburn's equation (Darcy, 1856; Washburn, 1921; Whitaker, 1986). However, based on the assumptions that the average pore size was large enough to enable a fast flow and that water could penetrate a short distance only due to the relatively short tablet height, an application of these models was found to be inadequate. Furthermore, any parameters relating to the tablet structure and the viscosity of water were considered to remain constant in section 1 and combined in a single kinetic constant for pore penetration. Therefore, a linear behaviour was considered to simplistically describe the water uptake in section 1 from the measurement start to the transition point was expressed according to Eq. 2:

(2) $$∆m\left(t\right)=k_{P}∙t\;\text{for}\;0\leq t\leq t^{*}$$

with water uptake Δm(t), kinetic constant for pore penetration k_P_, time t and transition point t*.

Secondly, the swelling of tablets was described according to Korsmeyer et al., who fitted the fractional drug release from swelling porous and hydrophilic polymers to a power law and determined a kinetic constant and an exponent for each drug-polymer system (Korsmeyer et al., 1983). Thus, the volume increase in section 2 dominated by swelling from the transition point to the end of the measurement was modelled with a power law, according to Eq. 3

(3) $$∆V\left(t\right)=k_{S}∙{\left(t-t^{*}\right)}^{n}\;\text{for}\;t>t^{*}$$

with volume increase ΔV(t), kinetic constant for swelling k_S_, swelling exponent n, time t and transition point t*.

Thirdly, the water uptake from the transition point to the end of the measurement was described by a combination of Eqs. (2), (3), according to Eq. 4:

(4) $$∆m\left(t\right)=k_{P}∙t^{*}+k_{S}∙{\left(t-t^{*}\right)}^{n}∙\rho_{w}\;\text{for}\;t>t^{*}$$

with water uptake Δm(t), kinetic constant for pore penetration k_P_, kinetic constant for swelling k_S_, swelling exponent n, density of water ρ_w_, time t and transition point t*.

The transfer of volume increase to water uptake was made by multiplication with the density of water, ρ_w_ = 1 mg/mm^3^ at a temperature of 22 ± 2 °C, considering an ideal mixture of water and tablet excipients. Thus, the excess volume was assumed to be negligible.

All measurement data were fitted to the empirical model to determine four characteristic parameters, namely the kinetic constants k_S_ and k_P_, the swelling exponent n and the transition point t*. The fitting operations of water uptake and swelling data from single tablets using Eqs. (2), (3), (4), were performed using the software OriginPro (OriginPro 2020, OriginLab, Northampton, USA).

In addition, a recalculation of the water uptake of section 1 until the transition point, which describes primarily the water penetrating into the tablet pores, was done using the kinetic constant for pore penetration and the time, according to

(5) $$∆m_{\text{calc}}\left(t\right)=k_{P}∙t\;\text{for}\;0\leq t\leq t^{*}$$

with calculated water uptake Δm_calc_(t), kinetic constant for pore penetration k_P_, time t and transition point t*.

Formally, this recalculated water uptake of section 1 is expressed in section 2, when swelling is dominating, as a constant value calculated at the transition point, according to

(6) $$∆m_{\text{calc}}\left(t\right)=k_{P}∙t^{*}\;\text{for}\;t>t^{*}$$

with calculated water uptake Δm_calc_(t), kinetic constant for pore penetration k_P_, time t and transition point t*.

If those assumptions made to set up the empirical model were found to be valid, a clearly separated consideration of the mechanistic processes of water uptake, namely pore penetration and tablet swelling, could be enabled. This separation was realised by calculating the amount of water penetrating into the tablet pore volume, without any pore volume enhancement by swelling, from the raw data of the measurement, according to

(7) $$∆m_{\text{pore}}\left(t\right)=∆m\left(t\right)-∆V\left(t\right)∙\rho_{w}$$

with water penetrating into the tablet pore volume Δm_pore_(t), water uptake Δm(t), volume increase ΔV(t), time t and density of water ρ_w_.

The density of water ρ_w_ was considered to be constant at 1.00 mg/mm^3^.

A theoretical value for the mass of water in the tablet pores in the state of a completely filled pore volume can be calculated from the total pore volume and the density of water according to

(8) $$∆m_{\text{pore},\mathrm{th}}=V_{\text{pore}}∙\rho_{w}$$

with mass of water in completely filled tablet pores Δm_pore_,_th_, total pore volume V_pore_ and density of water ρ_w_.

## 3 Results and discussion

### 3.1 Water uptake of polymer extrudates

**Fig. 2:**
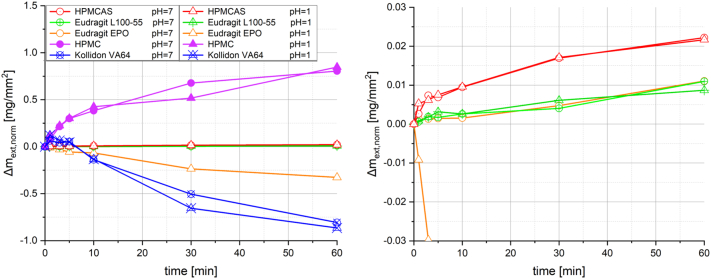
Normalised mass increase Δm_ext,norm_ of pure polymer extrudates immersed in water (*n* = 1); the right graph is an enlarged section of the left graph (focussing on the data for HPMCAS, Eudragit L100–55 and Eudragit EPO).

The normalised mass increase Δm_ext,norm_ of the polymer extrudates in water of different pH is shown in Fig. 2. HPMC showed the highest Δm_ext,norm_, with no influence of the pH. Δm_ext,norm_ of Kollidon VA64 increased similarly to HPMC during the first minute, which was followed by a fast decrease in the further course of the analysis, resulting in values below zero. For HPMCAS and Eudragit L100–55 extrudates, the Δm_ext,norm_ was approximately two orders of magnitude lower compared to HPMC extrudates. The values of HPMCAS were approximately twice as high as those of Eudragit L100–55, both with no impact of the pH. In contrast, for Eudragit EPO the pH influenced Δm_ext,norm_. At a pH of 7, a similar behaviour compared to Eudragit L100–55 was observed, whereas at a pH of 1 a continuous decrease of the mass was observed. HPMC seemed to strongly absorb water by diffusion under the formation of a relatively stable physical gel layer, without fast polymer dissolution. This might be explained by a certain motion of the polymer chains upon interaction with the water molecules. However, physical associations, such as hydrogen bonds, might still maintain the entangled state of the chains and prevent polymer dissolution (Katzhendler et al., 2000; Rubinstein and Colby, 2003). In contrast, Kollidon VA64 rapidly dissolved in the test medium, after a short initial phase of strong water absorption. Also during this experiment, a physical gel layer might have formed, which continuously moved from the surface towards the centre of the extrudates by approximately similar kinetics of diffusive water absorption and complete disentanglement of the polymer chains. As expected by the chemical composition of the polymers, no impact of the pH of the test medium on the swelling behaviour of HPMC and Kollidon VA64 was observed. Only a minimal water absorption and hence no gelling or dissolution was observed for HPMCAS and Eudragit L100–55 in the different test media, although both polymers contain acidic groups. Eudragit EPO showed a distinct pH sensitivity in this analysis, as a fast dissolution of the polymer containing basic groups could be noticed only in the test medium with a pH of 1. In contrast to HPMC and Kollidon VA64, no gelling was observed for Eudragit EPO, which might be explained by the low water absorption capacity.

### 3.2 Water uptake and swelling of tablets

#### 3.2.1 Pure polymer tablets

The characteristic particle sizes x_10,3_, x_50,3_ and x_90,3_ of the size distribution and specific surface areas S_m_ of polymer powders after milling used for the manufacturing of tablets are listed in Table 1.

**Table 1:** Characteristic particle sizes x_10,3_, x_50,3_ and x_90,3_ of the size distributions and specific surface areas S_m_ of pure polymer powders prior to tablet manufacturing.

Polymer	x_10,3_	x_50,3_	x_90,3_	S_m_
	[μm]	[μm]	[μm]	[m^2^/g]
HPMCAS	44	155	342	0.07
Eudragit L100–55	42	233	431	0.07
Eudragit EPO	62	203	364	0.05
HPMC	145	340	655	0.02
Kollidon VA64	27	177	379	0.09

**Fig. 3:**
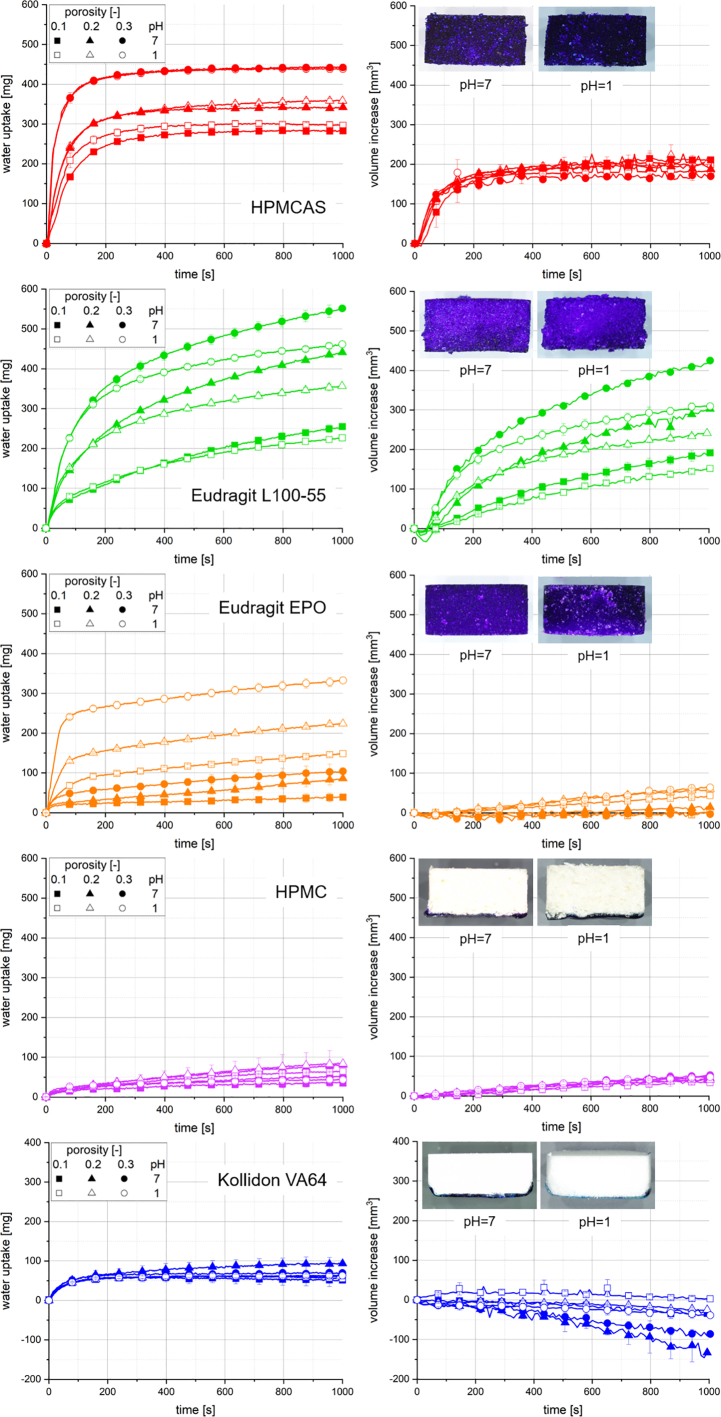
Water uptake (left) and volume increase (right) with standard deviation (*n* = 3) of pure polymer tablets at different pH; the line represents the connection of 7 data points per second for water uptake and 6 data points per minute for volume increase; microscope images of the cut surface of tablets with a porosity of 0.2 from the complementary water penetration analysis with dyed water at a pH of 7 (left) and a pH of 1 (right), respectively, after 1000 s. Please note the different scale of the data shown for Kollidon VA64 (bottom).

Fig. 3 shows the water uptake and swelling measurement results of the pure polymer tablets, as well as the images of the tablet cut surfaces obtained with dyed water at a pH of 1 and a pH of 7, respectively.

For HPMCAS, Eudragit L100–55 and Eudragit EPO tablets, both initial water uptake rate and total water uptake at the end of the measurement increased with increasing porosity. Furthermore, the results of Eudragit L100–55 and Eudragit EPO tablets were systematically impacted by the pH of the water, where the total water uptake of Eudragit L100–55 tablets was higher at a pH of 7 compared to a pH of 1. In contrast, significantly higher initial water uptake rates and total water uptake values were measured at a pH of 1 for Eudragit EPO tablets. Initial water uptake rates and total water uptake values of HPMC and Kollidon VA64 tablets were relatively low and no impact of porosity or pH was observed.

The volume increase of Eudragit L100–55 tablets increased with porosity and was higher at a pH of 7 compared to a pH of 1, which displayed a similar behaviour compared to the water uptake of these tablets. Eudragit L100–55 tablets with a porosity of 0.3 showed the highest total water uptake and volume increase throughout all measured samples. The swelling of HPMCAS and HPMC tablets was neither affected by the porosity nor the pH of the water. However, the volume increase of HPMC tablets was significantly lower compared to HPMCAS tablets and showed an approximately linear behaviour. A similar low and rather linear volume increase was observed for all Eudragit EPO tablets at a pH of 1. Contrarily, almost no swelling was observed at a pH of 7. For nearly all Kollidon VA64 tablets, a decrease of the volume was measured. This decrease was stronger for tablets with porosities of 0.2 and 0.3 compared to 0.1, as well as for the measurement at a pH of 7 compared to a pH of 1.

The microscope images showed that the dyed water penetrated HPMCAS, Eudragit L100–55 and Eudragit EPO tablets entirely. In contrast, a gel layer was observed at the bottom of the HPMC and Kollidon VA64 tablets, which inhibited the further penetration of the water.

Comparing the normalised water uptake of polymer extrudates (Fig. 2) and the simultaneously measured water uptake and swelling of polymer tablets (Fig. 3), different characteristics of the polymer types were found. In general, the analysis of non-porous polymer extrudates with a relatively low specific surface area provided information about the kinetics of water uptake of the polymer material by diffusion. Polymer powders with a relatively high specific surface area were manufactured and compacted to tablets with a certain pore volume. The water uptake of these tablets could be induced by both pore penetration and swelling, which could result from both the water uptake by diffusion and the release of stored mechanical energy upon compaction of the polymer particles. Hence, pore penetration is a prerequisite for an increase of the swelling rate, as it increases the total wetted surface area of the polymer tablets. HPMC extrudates showed by far the highest normalised water uptake, whereas the water uptake and volume increase of tablets was relatively low compared to other polymers. The microscope images revealed that a gel layer formed at the bottom of the HPMC tablets upon contact with the water, which is in accordance with the reported findings of Chen et al. (Chen et al., 2010). This gel layer probably inhibited further water penetration into the pores and hence extensive swelling of the tablets. Similarly, a gel layer was observed at the bottom of Kollidon VA64 tablets and a decreasing tablet volume was identified. This result was found to be in accordance with the analysis of the Kollidon VA64 extrudates, which showed a fast decreasing Δm_ext,norm_ due to dissolution. Δm_ext,norm_ of HPMCAS and Eudragit L100–55 extrudates was much lower compared to HPMC extrudates. In contrast, water uptake and volume increase of HPMCAS and Eudragit L100–55 tablets was much higher compared to HPMC tablets, due to the unimpeded pore penetration and hence wetting of the inner surface area, which enabled fast swelling rates.

The water uptake was found to increase for HPMCAS and Eudragit L100–55 tablets with increasing porosity. After approximately 800 s, HPMCAS tablets reached a constant water uptake and volume, which might indicate a complete water penetration into the pores, a saturation of the polymer particles with water and a release of potentially stored mechanical energy, if applicable. In addition, a complete pore penetration could be expected from the complementary water penetration analysis using the dyed water. In contrast, water uptake and volume of Eudragit L100–55 tablets was still increasing after a measurement duration of 1000 s. The complementary water penetration analysis showed that pore penetration had already been completed, whereas swelling seemed to be still in progress according to the analysis results. HPMCAS extrudates were found to swell faster compared to Eudragit L100–55 extrudates, which might support the hypothesis for HPMCAS tablets reaching the saturation state of water uptake and volume increase relatively fast compared to Eudragit L100–55 tablets. While there was no impact of the pH on the analysis results of HPMCAS and Eudragit L100–55 extrudates, Eudragit L100–55 tablets were found to have higher water uptake and volume increase at a pH of 7 compared to a pH of 1. This pH sensitivity can be explained by the presence of free carboxyl groups in Eudragit L100–55. However, for HPMCAS, which also contains free carboxyl groups, a pH sensitivity could neither be detected for extrudates nor for tablets in this study. This might be related to the amount of free carboxyl groups per mass unit, which is approximately three times higher for Eudragit L100–55 compared to HPMCAS (Hughey et al., 2010). The pH sensitivity of Eudragit EPO, which is a polymer containing free aminoalkyl groups, was found to have a major impact on the normalised water uptake of extrudates, as well as the water uptake and swelling of tablets. Eudragit EPO extrudates showed a very low Δm_ext,norm_ at a pH of 7, similar to Eudragit L100–55, whereas at a pH of 1 a fast decrease was observed due to polymer dissolution. Further, a relatively low and approximately linear water uptake over the entire measurement period and practically no volume increase was measured for Eudragit EPO tablets at a pH of 7. Galeano et al. reported a poor wetting behaviour of Eudragit EPO with water, which might explain the slow water uptake, possibly due to the inhibition of water penetration into smaller pores (Galeano et al., 2019). At a pH of 1, water uptake was significantly higher and faster, which might indicate improved wetting of the polymer surface area leading to faster pore penetration. Interestingly, a low and approximately linear volume increase was determined in contrast to the extrudates at a pH of 1, which might be explained by the release of stored mechanical energy in the tablets.

Further, the results allow an interesting classification of the five polymers regarding their chemical composition. Low water uptake and volume increase due to the formation of a gel layer was measured only for the neutral polymers, which do not contain any acidic or basic groups. No gel formation was observed for the pH sensitive polymers. Intermolecular interactions between water and the polymer might lead to local changes of the pH at the polymer surface, possibly slowing down the absorption of the water. Therefore, gel formation might not noticeably impede water uptake and volume increase of the tablets. Tablets containing polymers which are prone to form a gel might also show slower disintegration and API dissolution behaviour. In general, such different characteristics of the polymers and their potential impact on the disintegration and dissolution behaviour should be considered during tablet formulation development.

The comparison of these results obtained on different test specimens of the same polymer underlines the importance of analytical methods on tablets, such as the simultaneous water uptake and swelling analysis, for the investigation of the tablet performance. The structural properties of the test specimens, such as shape and porosity, have a major impact on the processes and must be considered.

#### 3.2.2 Multi-components tablets

Water uptake and swelling of pure Kollidon VA64 tablets were very low due to the formation of a gel layer and partial dissolution. Therefore, Kollidon VA64 was selected for further investigations into multi-component tablets, as the water uptake and swelling of those tablets were expected to be significantly influenced by the addition of suitable excipients.

**Fig. 4:**
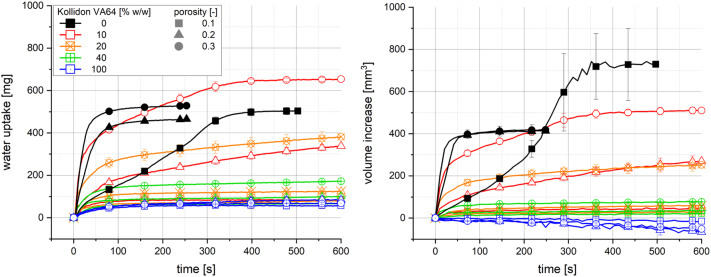
Water uptake (left) and volume increase (right) with standard deviation (n = 3) of tablets containing Kollidon VA64 and MCC in different proportions; the line represents the connection of 7 data points per second for water uptake and 6 data points per minute for volume increase.

The impact of polymer content and porosity of tablets containing MCC and Kollidon VA64 on water uptake and swelling are displayed in Fig. 4. The analysis results of pure MCC tablets (0% Kollidon VA64) were reported in an earlier study (Lenz et al., 2021). Only tablets with 10% Kollidon VA64 and a porosity of 0.3 showed a similar initial water uptake rate and an even higher total water uptake and volume increase compared to pure MCC tablets with a porosity of 0.3. An increase of Kollidon VA64 to 20% or a decrease of the tablet porosity to 0.2 resulted in a noticeable inhibition of water uptake and swelling of the tablets. Tablets containing 40% Kollidon VA64 showed a relatively low total water uptake, with results of tablets with porosities of 0.2 and 0.1 being comparable with pure Kollidon VA64 tablets. Further, the measured volume increase of these tablets was similarly low. However, no volume decrease was observed as in the case of pure Kollidon VA64. The drastically inhibited water uptake and swelling of tablets with polymer content above 20% and porosities below 0.2 might be explained by the tendency of Kollidon VA64 to form a gelling polymer network (GPN), hindering the water to penetrate into the tablet pores, as observed for pure Kollidon VA64 tablets and extrudates. This process of GPN formation probably starts with strong water absorption of the polymer particles, followed by fast partial dissolution. With the assumptions that the polymer particles are connected by the pore system within the tablet and that the water supply through the pores is relatively low, it might be conceived that the motion of the polymer chains within the pores is drastically limited, hence forming a network of a viscoelastic gel. Interestingly, the water uptake and volume increase of tablets with 10% Kollidon VA64 and a porosity of 0.3 was even higher compared to those with pure MCC. In this case, the dispersed Kollidon VA64 particles might have formed gel droplets only in a microenvironment, and water could still penetrate the entire tablet due to the high amount of the hydrophilic and swelling filler. Moreover, high water absorption of the Kollidon VA64 particles might even provoke a disintegrant-like behaviour in such a tablet formulation.

DiNunzio et al. reported gel formation in tablets based on Kollidon VA64 and MCC with a polymer content above 50%, resulting in incomplete disintegration and hindered dissolution. However, these tablets contained 10% *w*/w NaCMCXL (DiNunzio et al., 2012). Goddeeris et al. found acceptable disintegration times of tablets containing at least 75% MCC and an ASD based on Kollidon VA64 and d-α-tocopheryl polyethylene glycol succinate 1000. In this formulation, PVPP was used as disintegrant representing 25% w/w of the ASD (Goddeeris et al., 2008). Dong et al. reported an inhibition of water penetration and hence lower swelling of tablets containing a solid dispersion based on HPMC due to the formation of a gel layer. High amounts of MCC promote tablet hydration, as the material swells more rapidly and no gelling occurs (Dong et al., 2021). This might be in good agreement with the comparable behaviour of the investigated tablet formulations containing Kollidon VA64 and MCC displayed in Fig. 4. The analytical method employed in this study provides additional quantitative information about the performance of tablets regarding gel forming ingredients, leading to the inhibition of disintegration and dissolution reported in the above-mentioned publications.

**Fig. 5:**
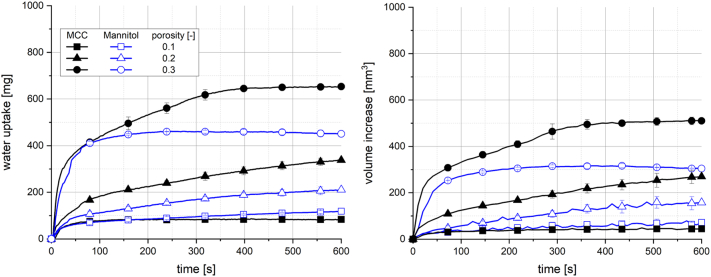
Water uptake (left) and volume increase (right) with standard deviation (n = 3) of tablets containing 10% Kollidon VA64 and fillers MCC and Mannitol; the line represents the connection of 7 data points per second for water uptake and 6 data points per minute for volume increase.

Fig. 5 shows a comparison of the fillers MCC and mannitol in a binary formulation with 10% Kollidon VA64. Total water uptake and volume increase were higher for tablets containing MCC with porosities of 0.2 and 0.3. Interestingly, tablets based on mannitol with a porosity of 0.1 showed a slightly higher total water uptake and volume increase compared to those based on MCC. The maximum value of water uptake and volume increase for tablets containing mannitol with a porosity of 0.3 was observed between 300 and 400 s, followed by a slight and continuous decrease of both quantities towards the end of the measurement. This decrease might indicate dissolution, as mannitol is a water-soluble filler. This might explain that tablets containing water-insoluble filler MCC showed an enhanced water uptake and swelling capacity compared to water-soluble mannitol.

**Fig. 6:**
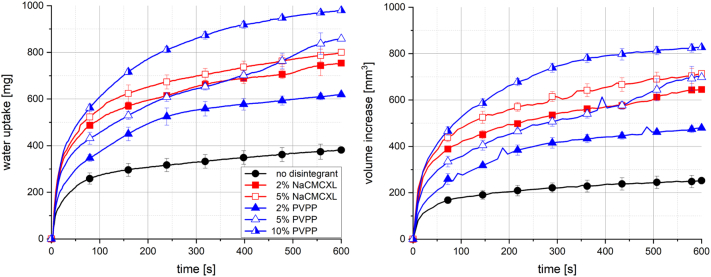
Water uptake (left) and volume increase (right) with standard deviation (n = 3) of tablets containing 20% Kollidon VA64, MCC and disintegrants NaCMC and PVPP with constant porosity of 0.3; the line represents the connection of 7 data points per second for water uptake and 6 data points per minute for volume increase.

In Fig. 6, the disintegrants NaCMCXL and PVPP are compared in a tablet formulation based on MCC and 20% Kollidon VA64 with a porosity of 0.3. In general, a noticeable enhancement of total water uptake and swelling was observed upon the addition of disintegrants. However, the very initial water uptake rate was not affected. The total water uptake of tablets containing PVPP could be increased with increasing disintegrant content. Water uptake and swelling was only slightly enhanced by the increase of disintegrant content from 2% to 5% for tablets containing NaCMCXL. The total water uptake and volume increase was higher for NaCMCXL compared to PVPP, when the disintegrant content was 2%. The presented results show that 2% NaCMCXL lead to a stronger increase of water uptake and volume increase of tablets compared to 2% PVPP. This was found to be in accordance with the findings of Quodbach and Kleinebudde (Quodbach and Kleinebudde, 2014). Further, a disintegrant content of 5% resulted in a noticeable increase of water uptake and volume increase for PVPP compared to 2%, whereas for NaCMCXL only a small increase was observed. Moreover, approximately similar results were measured for 5% PVPP as for 2% and 5% NaCMCXL, which might be explained by the tendency of NaCMCXL to form a GPN, limiting the water uptake and swelling at high disintegrant content (Haware et al., 2008; Desai et al., 2014). Water uptake and swelling of tablets might further be related to their disintegration behaviour (Markl et al., 2021). Zhao and Augsburger reported comparable disintegration times of tablets containing NaCMCXL and PVPP in equal proportion (Zhao and Augsburger, 2005). Moreover, they measured a higher increase of maximal tablet water uptake with increasing disintegrant content for PVPP compared to NaCMCXL. However, they used a PVPP quality with a smaller particle size compared to the material used in this study.

**Fig. 7:**
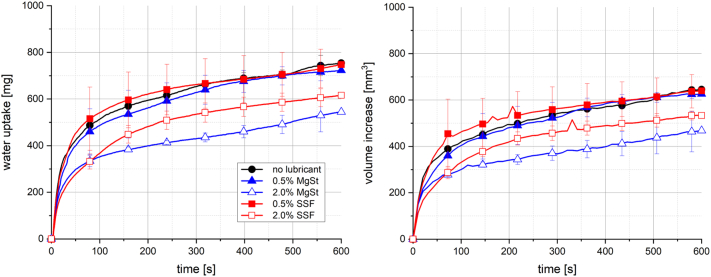
Water uptake (left) and volume increase (right) with standard deviation (n = 3) of tablets containing 20% Kollidon VA64, MCC, 2% NaCMCXL and lubricants MgSt and SSF with constant porosity of 0.3; the line represents the connection of 7 data points per second for water uptake and 6 data points per minute for volume increase.

The impact of the lubricants MgSt and SSF on water uptake and swelling is illustrated in Fig. 7. No differences were observed for tablets with a lubricant content of 0.5% compared to those without lubricant. When the lubricant content was increased to 2%, the total water uptake and volume increase were inhibited noticeably. Further, the initial water uptake rates decreased slightly. These effects were stronger for tablets containing MgSt compared to those with SSF. These results are in good agreement with the findings of Late et al., who reported extended disintegration times of tablets containing hydrophobic MgSt (Late et al., 2009). The results of the present study might indicate the hydrophobic nature of both lubricants, leading to a decrease of the tablet wettability. As a consequence, reduced pore penetration and swelling could be observed, which is in accordance with the findings of Van Kamp et al. (Van Kamp et al., 1986). Due to this inhibition of these processes, a certain impact of disintegration or dissolution might be expected.

**Fig. 8:**
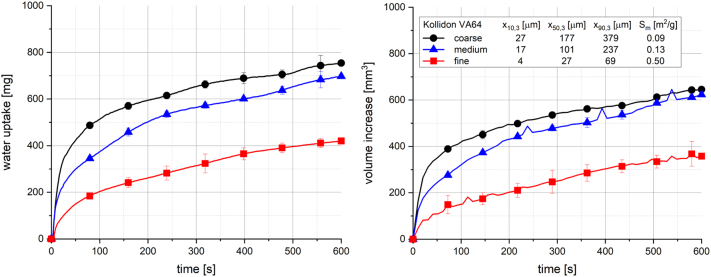
Water uptake (left) and volume increase (right) with standard deviation (n = 3) of tablets containing 20% Kollidon VA64 with different particle sizes, 78% MCC and 2% NaCMCXL with constant porosity of 0.3; the line represents the connection of 7 data points per second for water uptake and 6 data points per minute for volume increase.

Fig. 8 shows the influence of the particle size and specific surface area of Kollidon VA64 within a three-component tablet formulation on water uptake and swelling. Smaller particle size and higher specific surface area resulted in a decrease of the initial water uptake rate as well as the total water uptake and volume increase. The formation of a GPN was found to be the cause of the observed inhibition of water uptake and swelling for tablets containing Kollidon VA64. Moreover, this GPN formation might be supported by decreasing particle size of the Kollidon VA64 due to the higher specific surface area of the polymer, the probably smaller pore sizes and the finely branched network of the polymer particles within these tablets.

#### 3.2.3 Modelling of water uptake and swelling

**Fig. 9:**
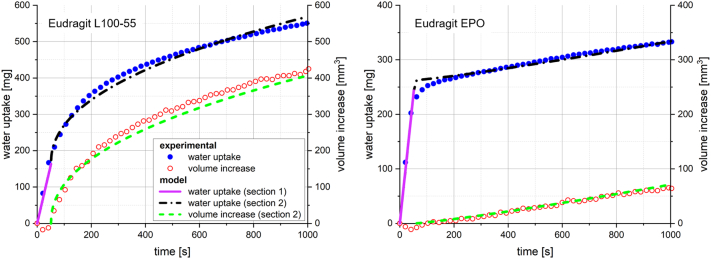
Experimental and fitted water uptake and swelling (n = 3) according the developed empirical model for Eudragit L100–55 with a porosity 0.3 at a pH of 7 (left) and Eudragit EPO with a porosity 0.3 at a pH of 1 (right).

Modelling results for water uptake and swelling of Eudragit L100–55 with a porosity of 0.3 at a pH of 7 and Eudragit EPO with a porosity of 0.3 at a pH of 1 are compared with the experimentally obtained data in Fig. 9. Each of the three sections of the empirical model is separately illustrated. The fitted curves were found to describe the measured data adequately for both examples, depicting the significantly different water uptake and swelling behaviour of the tablets.

Characteristic parameters of selected experiments determined by data fitting and the average coefficients of determination (R^2^) are given in Table 2, in order to demonstrate the ability of the model to discriminate between different water uptake and swelling profiles. These values describe the average of each characteristic parameter being determined for every single analysed tablet (*n* = 3). Thus, the presented standard deviation includes any deviation, including measurement errors, variations of tablet samples and deviations of the fitting procedure itself. Further, characteristic parameters of all other experiments are provided in a supplement file.

**Table 2:** Characteristic parameters (average and standard deviation) determined by fitting of water uptake and swelling data of selected experiments according to the empirical model using Eqs. (2), (3), (4): kinetic constant for pore penetration k_P_, kinetic constant for swelling k_S_, swelling exponent n and transition point t*, as well as the average coefficient of determination (R^2^). The standard deviation includes any deviation, including tablet manufacturing variations, measurement errors from water uptake and swelling analysis, and deviations from the mathematical fitting procedure.

Formulation	Composition	Porosity	pH	k_P_	k_S_	n	t*	R^2^
	[% w/w]	[−]	[−]	[mg/s]	[mm^3^/s^n^]	[−]	[s]	[−]
HPMCAS	100	0.1	7	1.45 ± 0.25	65.36 ± 20.12	0.18 ± 0.04	58 ± 5	0.970
HPMCAS	100	0.2	7	3.51 ± 0.11	78.17 ± 2.35	0.14 ± 0.00	41 ± 0	0.980
HPMCAS	100	0.3	7	10.05 ± 0.90	66.41 ± 3.38	0.15 ± 0.01	27 ± 3	0.991
Eudragit L100–55	100	0.1	7	0.88 ± 0.11	2.54 ± 0.96	0.64 ± 0.04	76 ± 4	0.995
Eudragit L100–55	100	0.2	7	1.77 ± 0.04	8.66 ± 0.48	0.52 ± 0.01	81 ± 1	0.994
Eudragit L100–55	100	0.3	7	2.69 ± 0.21	26.32 ± 2.05	0.40 ± 0.01	56 ± 3	0.993
Eudragit EPO	100	0.1	1	0.93 ± 0.23	0.03 ± 0.01	1.08 ± 0.09	111 ± 22	0.992
Eudragit EPO	100	0.2	1	2.01 ± 0.17	0.08 ± 0.04	1.03 ± 0.11	78 ± 8	0.995
Eudragit EPO	100	0.3	1	4.90 ± 0.19	0.04 ± 0.02	1.11 ± 0.08	54 ± 3	0.998
Kollidon VA64	100	0.1	7	0.69 ± 0.33	−1.16 ± 1.30	0.60 ± 0.24	129 ± 57	0.970
Kollidon VA64	100	0.2	7	0.62 ± 0.10	−20.58 ± 4.45	0.21 ± 0.08	243 ± 37	0.892
Kollidon VA64	100	0.3	7	0.57 ± 0.11	−11.20 ± 3.58	0.27 ± 0.04	219 ± 43	0.934
HPMC	100	0.1	7	0.10 ± 0.14	0.23 ± 0.14	0.77 ± 0.09	21 ± 30	0.834
HPMC	100	0.2	7	0.44 ± 0.24	0.31 ± 0.10	0.76 ± 0.06	85 ± 54	0.980
HPMC	100	0.3	7	0.35 ± 0.47	0.32 ± 0.08	0.71 ± 0.06	35 ± 23	0.919
Kollidon VA64/MCC	20/80	0.3	7	11.57 ± 1.63	56.81 ± 8.39	0.24 ± 0.02	9 ± 1	0.983
Kollidon VA64/MCC/NaCMCXL	20/78/2	0.3	7	12.53 ± 2.46	140.24 ± 24.95	0.24 ± 0.03	9 ± 1	0.990
Kollidon VA64/MCC/NaCMCXL	20/75/5	0.3	7	10.55 ± 1.59	168.85 ± 18.82	0.23 ± 0.02	8 ± 1	0.990
Kollidon VA64/MCC/PVPP	20/78/2	0.3	7	14.63 ± 0.48	65.24 ± 6.54	0.32 ± 0.01	9 ± 1	0.975
Kollidon VA64/MCC/PVPP	20/75/5	0.3	7	18.41 ± 9.14	92.21 ± 27.99	0.33 ± 0.02	6 ± 2	0.990
Kollidon VA64/MCC/PVPP	20/70/10	0.3	7	12.97 ± 2.95	152.91 ± 2.98	0.27 ± 0.00	9 ± 0	0.987

**Fig. 10:**
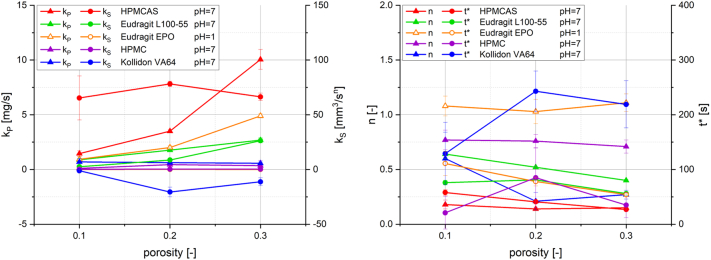
Characteristic parameters determined by fitting of water uptake and swelling data as function of tablet porosity: kinetic constant for pore penetration k_P_ and kinetic constant for swelling k_S_ (left), swelling exponent n and transition point t* (right). The data describe selected experiments with pure polymer tablets. The standard deviation includes any deviation, including tablet manufacturing variations, measurement errors from water uptake and swelling analysis, and deviations from the mathematical fitting procedure.

The characteristic parameters k_P_ and k_S_ (left) as well as n and t* (right) of the selected pure polymer tablets are plotted as a function of porosity for a better visualization in Fig. 10.

Pure HPMCAS tablets showed a strongly increasing kinetic constant for pore penetration k_P_ and a decreasing transition point t* with increasing porosity, whereas the kinetic constant for swelling k_S_ and the swelling exponent n were approximately comparable at a pH of 7. k_P_ increased less strongly with increasing porosity for Eudragit L100–55 tablets at a pH of 7 and t* was slightly higher compared to HPMCAS. However, significantly increasing values of k_S_ and decreasing values of n were determined for higher tablet porosities for Eudragit L100–55 tablets. Eudragit EPO tablets showed a moderately increasing k_P_ and decreasing t* with increasing porosity. k_S_ was found to be relatively low compared to HPMCAS and Eudragit L100–55, whereas the values of the swelling exponent n were slightly above 1, indicating a nearly linear swelling behaviour. Both k_P_ and k_S_ are relatively low for HPMC tablets, whereas n is relatively high. Kollidon VA64 tablets similarly showed relatively low k_P_ and the highest t* values of all tablets. The k_S_ values were negative for all porosities.

Except for Eudragit L100–55 and Kollidon VA64, n remained nearly constant for the other polymers and all porosities.

The increasing initial water uptake with increasing porosity being observed for those pure polymer tablets of HPMCAS, Eudragit L100–55 and Eudragit EPO, was found to be well described by the increasing values of the kinetic constant for pore penetration k_P_. In addition to k_P_, the transition point t* needs to be considered as well to interpret the first measurement section, where pore penetration is dominant. Further, the impeded water uptake, or pore penetration in particular, caused by GPN formation in HPMC and Kollidon VA64 was expressed by low k_P_ values. The volume increase of HPMCAS tablets was similar for all porosities, which could be recognised by similar values of the kinetic constant for swelling k_S_ and the swelling exponent n. In contrast, the volume of Eudragit L100–55 tablets increased noticeably more strongly with increasing porosity, which resulted in highly increasing values of k_S_ and decreasing values of the swelling exponent n. The swelling exponent always needs to be considered in combination with k_S_ for the complete characterisation of the swelling behaviour. This can be further observed in the characterisation of Eudragit EPO tablets at a pH of 1, where only a low and nearly linear volume increase was measured. The determined values of k_S_ were significantly lower compared to HPMCAS and Eudragit L100–55 at a pH of 7 and the swelling exponent n was slightly above 1. The low volume increase of HPMC tablets was described by relatively low values of k_S_ and relatiely high values of n of approximately 0.75 for all porosities. The comparison of k_S_ and n between Eudragit EPO at a pH of 1 and HPMC at a pH of 7 is an example showing how the model enables a more precise and quantitative discrimination of the two samples with an apparently similar swelling behaviour. The k_S_ values for Kollidon VA64 tablets were negative for all porosities. Although this might qualitatively indicate a partial dissolution of the polymer, an interpretation of such negative values should be done very carefully, as the theoretical assumptions of the model do not include dissolution processes.

**Fig. 11:**
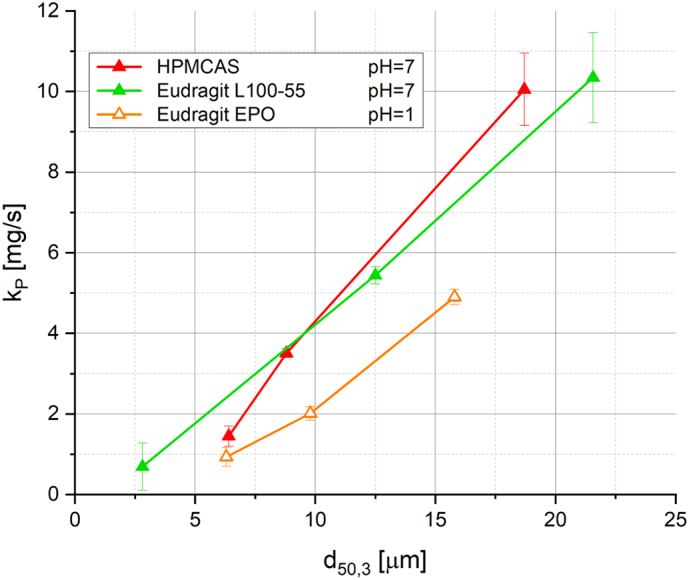
Kinetic constant for pore penetration k_P_ determined by fitting of water uptake and swelling data as function of tablet pore size d_50,3_. The data describe selected experiments with pure polymer tablets. The standard deviation includes any deviation, including tablet manufacturing variations, measurement errors from water uptake and swelling analysis, and deviations from the mathematical fitting procedure.

Fig. 11 shows the kinetic constant for pore penetration k_P_ of pure HPMCAS and Eudragit L100–55 tablets at a pH of 7, and pure Eudragit EPO tablets at a pH of 1, as a function of the average pore size d_50,3_ determined by mercury porosimetry. For these polymers, a nearly linear relation of k_P_ and d_50,3_ was observed. This behaviour might even be anticipated after a theoretical consideration with the simplifying assumption that all tablets consist of an equal number of cylindrical capillaries. The capillary pressure, which is driving the pore penetration, is inversely proportional to the capillary diameter, and the volume of the capillary is proportional to the square of the capillary diameter. Further, the water uptake rate in the phase where pore penetration is the dominant mechanism (section 1) was considered to be constant. These two considerations might explain such a linear relation of k_P_ and d_50,3_. However, only a few data sets were taken into account. To further support this hypothesis, more experiments with a focus on the tablet pore size would be required.

**Fig. 12:**
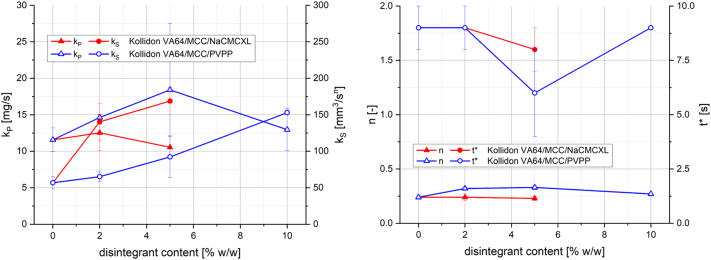
Characteristic parameters determined by fitting of water uptake and swelling data as function of disintegrant content: kinetic constant for pore penetration k_P_ and kinetic constant for swelling k_S_ (left), swelling exponent n and transition point t* (right). The data describe selected experiments with tablets containing 20% Kollidon VA64 and varying proportions of MCC and disintegrant. The standard deviation includes any deviation, including tablet manufacturing variations, measurement errors from water uptake and swelling analysis, and deviations from the mathematical fitting procedure.

Similar to the pure polymer tablet experiments, the characteristic parameters, k_P_ and k_S_ (left), as well as n and t* (right) of the selected experiments with tablets containing Kollidon VA64, MCC and disintegrant are plotted as a function of disintegrant content in Fig. 12.

NaCMCXL did not have a substantial impact on k_P_, n and t* of tablets with a porosity of 0.3 containing 20% Kollidon VA64 and 78 or 75% MCC, respectively. However, k_S_ increased drastically with a higher NaCMCXL content. In contrast, PVPP induced a noticeable increase of k_P_ compared with the tablets without disintegrant, which was maximal at a disintegrant content of 5%. Additionally, a minimal value of t* was observed for this specific formulation. The swelling exponent n was higher when PVPP was used instead of NaCMCXL, which was more pronounced at lower disintegrant content. Further, k_S_ increased with PVPP content, however, the absolute values were lower than those of tablets with similar NaCMCXL content. The value of k_S_ was lower for tablets with 2% PVPP compared to those without any disintegrant. In general, the transition point was determined to be within the first 10 s of the measurements of tablets based on 20% Kollidon VA64, MCC and varying disintegrant content with a porosity of 0.3.

There were no major differences of initial water uptake ascertained for the tablets containing Kollidon VA64, MCC and varying disintegrant content, and therefore the values of k_P_ were similar.

Tablets containing Kollidon VA64, MCC and NaCMCXL showed a strongly enhanced swelling compared to formulations without disintegrant, which could be further ascertained with significantly higher k_S_ values of tablets at constant n. Formulations with PVPP were found to have a higher volume increase with an increasing amount of disintegrant. k_S_ increased consistently for 2%, 5% and 10% disintegrant. However, the value was slightly higher for tablets without disintegrant compared to tablets containing 2% PVPP. For this comparison, the increased swelling exponent of the formulations with PVPP needs necessarily to be considered. In general, the transition point t* of the measurements with tablets containing Kollidon VA64, MCC and disintegrants was determined to be much earlier compared to the pure polymer tablets, which might be explained by the high amount of fast and extensively swelling excipients MCC, NaCMCXL and PVPP.

The swelling exponent n was nearly constant for tablets with similar composition for all porosities, except Eudragit L100–55, where a moderate decrease of n with increasing porosity was observed. Therefore, if such a behaviour of constant n is observed in future experiments, the swelling exponent might be considered as material characteristic, as done to describe diffusion-controlled API release kinetics in polymer compacts by Korsmeyer et al. (Korsmeyer et al., 1983).

**Fig. 13:**
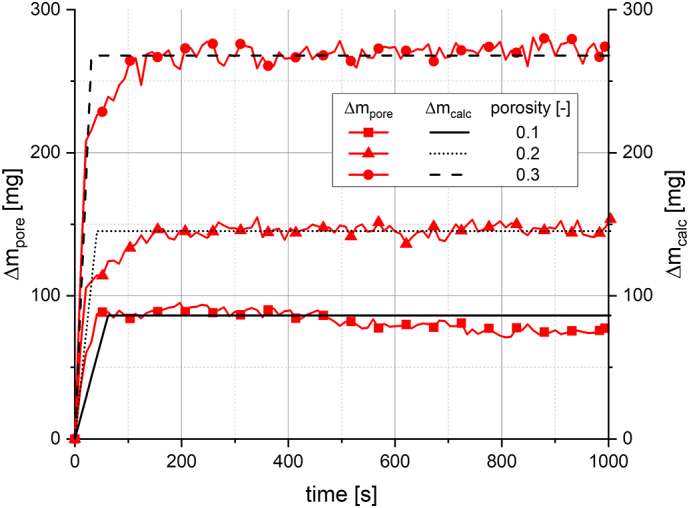
Water penetrated into the tablet pore volume Δm_pore_(t) and calculated water uptake Δm_calc_(t) for pure HPMCAS tablets at a pH of 7 (n = 3).

Fig. 13 displays Δm_pore_ for the analysis of HPMCAS tablets at a pH of 7 (red data points and lines), which was determined by the difference of water uptake data and the volume increase data multiplied by the density of water, according to Eq. 7. If the assumptions made to set up the model are valid, Δm_pore_ describes the water penetrating into the tablet pore volume. Additionally, Δm_calc_, which is the recalculated water uptake using k_P_ (black lines) is shown in Fig. 13. For all tablet porosities, Δm_pore_ converged towards a constant value after approximately 150 s. This constant value increased with increasing tablet porosity, which might indicate that the test medium penetrated into the entire pore volume of those tablets within this time period. Δm_calc_ was approximately equal to Δm_pore_ for *t* ≥ 150 s. This showed that the water penetrating into the tablet pores might also be estimated by a calculation of Δm_calc_ from the determined parameters k_P_ and t*. With the comparison of Δm_calc_ and Δm_pore_ for t ≤ t*, the assumption of a linear water uptake mainly by pore penetration in section 1 was found to be reasonable for HPMCAS tablets. For those tablets with a porosity of 0.2 and 0.3, Δm_calc_ was approximately equal to Δm_pore_. The transition of Δm_pore_ from the linear increase to the constant value might be explained by a rather complex pore structure of the tablets. Thus, the penetrating test medium might not have reached the top face of the tablets simultaneously in any radial position. For HPMCAS tablets with a porosity of 0.1, the increase of Δm_pore_ was slightly stronger compared to Δm_calc_. This might occur due to the slight initial volume decrease measured for those tablets (Fig. 3), which was not considered in the model and, therefore, also not in Δm_calc_.

Table 3 provides a comparison of Δm_calc_(t*) and the theoretical mass of water in completely filled tablet pores Δm_pore,th_, calculated according to Eq. 8, for the analysis of HPMCAS tablets at a pH of 7. It could be noted that Δm_calc_(t*) was 35 ± 3% higher compared to Δm_pore,th_ for all tablets with different porosity. This might indicate for those examples that at t* the water penetrated nearly completely into the initial pore volume. However, the noticeable deviation between Δm_calc_(t*) and Δm_pore,th_ must be considered and explained. There are several potential sources of error leading to the deviation of the values, including measurement errors of the water uptake and swelling data or the tablet porosity, or assumptions made to set up the model, such as the neglection of the excess volume upon swelling, and the hypothesis of the determination of pore penetration from the analysis data. In consideration of the high number of potential uncertainties, the hypothesis of describing the pore penetration by Δm_pore_ and Δm_calc_ was considered to be acceptable. However, the accuracy of the quantities was affected, and such deviations must be considered for the interpretation of the data. In particular, the excess volume of polymers upon swelling might be further investigated to understand and potentially minimise those deviations.

**Table 3:** Water uptake at transition point Δm_calc_(t*), recalculated according to Eq. 5, compared with the theoretical mass of water in completely filled tablet pores Δm_pore_,_th_ according to Eq. 8, for HPMCAS tablets at a pH of 7.

Formulation	Porosity	Δm_calc_(t*)	Δm_pore,th_
	[−]	[mg]	[mg]
HPMCAS	0.1	85	64
HPMCAS	0.2	145	104
HPMCAS	0.3	268	201

The aim of the developed empirical model was to improve the evaluation of water uptake and swelling behaviour of different tablets by the determination of four characteristic parameters. The model was based on rather simple equations describing water uptake and swelling, which was applied to fit the analysis data from two separate data sets of tablet water uptake and volume increase, respectively. Accordingly, no additional parameters of the tablet or the materials were required. In contrast, the model of Markl et al. focused in detail on the interacting processes of liquid penetration and swelling by combining two theoretical approaches, modelling and predicting the water penetration and axial swelling of tablets which are radially restricted (Markl et al., 2017). However, if tablets are not radially restricted during swelling, as it was the case in the experimental procedures of this study, the mechanistic processes are more complex and diverse. A generalisation of these complex processes and their subprocesses is very challenging, especially for multi-component industrial relevant tablet formulations. The newly presented modelling approach in this study neither considered any detailed considerations of solid, liquid or semi-solid physicochemical parameters of individual components or mixtures, nor could it be used for a priori predictions of water uptake and swelling behaviour. However, the new model was developed with the motivation for the application of industrial relevant, multi-component tablet formulations, in order to quantitatively discriminate their water uptake and swelling behaviour with four easily interpretable characteristic model parameters. The characteristic parameters were found to adequately describe the occurring simultaneous processes for the presented examples, fostering the quantitative analysis, comparison of tablet formulation properties and understanding of pore penetration and swelling. Further, this showed that the simplified linear model for initial pore penetration was adequate to describe the process, which could be considered as a linearisation of Darcy's law or Washburn's equation applied for the water transport over a short distance.

## 4 Conclusions

A systematic study for the tablet formulation development by water uptake and swelling analysis was performed. The main focus of the study concentrated on pharmaceutical polymers, which are commonly used as API carriers in amorphous solid dispersions (ASDs). The impact of formulation parameters, e.g. polymer type and particle size, filler, disintegrant, lubricant and tablet porosity, as well as the pH of the water could be clearly discriminated. The comparison of the analysis of water uptake and swelling of tablets with the analysis of normalised mass increase of polymer extrudates revealed the necessity of this analytical method, which includes the impact of the inherent structural properties of the tablets. These processes were considered as prerequisite for disintegration and, therefore, might have a distinct impact on the dissolution rate. The results showed that the type of used excipients, such as pharmaceutical polymers, fillers, disintegrants and lubricants and their proportions in the tablet formulation, as well as polymer particle size, pH of the test medium and tablet porosity could determine the extent of water uptake and swelling. Especially the formation of a gelling polymer network (GPN), which was observed throughout all HPMC and Kollidon VA64 tablets, lead to a strong inhibition of water penetration and swelling in the tablets and might be responsible for longer disintegration times and lower dissolution rates of the tablets. When these polymers are used, a high amount of additional excipients, such as fillers and disintegrants are crucial. Experimental data were fitted to an empirical model, describing the interacting processes of water uptake and volume increase. The four determined characteristic parameters could be used to quantitatively characterise and assess tablet components, compositions and inherent structural properties of different tablet formulations. Moreover, the analysis data and the model enabled a discriminative analysis of the simultaneous processes of pore penetration and swelling, which could provide a deeper understanding about complex material characteristics in pure and multi-component tablets. Therefore, the analysis of water uptake and swelling could be further compared with disintegration and dissolution data, to quantitatively ascertain and understand the functional relationship of tablet formulation and performance. The analytical methodology and the empirical model may also be used for conventional immediate-release or modified-release tablets with rotationally symmetrical shape, e.g. biconvex round, or mirror symmetrical shape, e.g. biconvex oblong.

## Declaration of Competing Interest

The authors declare that they have no known competing financial interests or personal relationships that could have appeared to influence the work reported in this paper.

## Appendix A Supplementary data

Supplementary material Image 1
