# Supplementary material for: Tablet formulation development focusing on the functional behaviour of water uptake and swelling
Source: Int J Pharm X. 2021 Nov 2;3:100103. doi: 10.1016/j.ijpx.2021.100103 (PMC8581513; doi:10.1016/j.ijpx.2021.100103)
Supplement: Supplementary file 1 — Supplementary material [file mmc1.docx]

| **formulation** | **composition** | **porosity** | **pH** | **k_P_** | **k_S_** | **n** | **t*** |
| --- | --- | --- | --- | --- | --- | --- | --- |
|  | [% w/w] | [-] | [-] | [mg/s] | [mm^3^/s^n^] | [-] | [s] |
| HPMCAS | 100 | 0.1 | 1 | 2.52 ± 0.48 | 78.71 ± 5.68 | 0.14 ± 0.01 | 45 ±10 |
| HPMCAS | 100 | 0.2 | 1 | 3.52 ± 0.07 | 73.22 ± 8.86 | 0.16 ± 0.02 | 41 ± 0 |
| HPMCAS | 100 | 0.3 | 1 | 11.12 ± 0.21 | 72.74 ± 1.30 | 0.14 ± 0.00 | 23 ± 1 |
| Eudragit L100-55 | 100 | 0.1 | 1 | 1.12 ± 0.07 | 1.42 ± 0.29 | 0.69 ± 0.02 | 73 ± 5 |
| Eudragit L100-55 | 100 | 0.2 | 1 | 1.98 ± 0.02 | 15.34 ± 0.77 | 0.40 ± 0.00 | 60 ± 0 |
| Eudragit L100-55 | 100 | 0.3 | 1 | 2.77 ± 0.07 | 34.69 ± 3.27 | 0.32 ± 0.01 | 58 ± 5 |
| Eudragit EPO | 100 | 0.1 | 7 | 0.59 ± 0.13 | 0.00 ± 0.00 | - | 68 ± 13 |
| Eudragit EPO | 100 | 0.2 | 7 | 0.44 ± 0.24 | 0.00 ± 0.00 | - | 264 ± 182 |
| Eudragit EPO | 100 | 0.3 | 7 | 0.51 ± 0.25 | 0.00 ± 0.00 | - | 225 ± 123 |
| Kollidon VA64 | 100 | 0.1 | 1 | 0.80 ± 0.30 | 0.35 ± 3.31 | 0.17 ± 0.18 | 127 ± 114 |
| Kollidon VA64 | 100 | 0.2 | 1 | 0.65 ± 0.11 | -5.20 ± 2.01 | 0.15 ± 0.06 | 112 ± 23 |
| Kollidon VA64 | 100 | 0.3 | 1 | 0.83 ± 0.18 | -13.29 ± 9.90 | 0.14 ± 0.10 | 102 ± 15 |
| HPMC | 100 | 0.1 | 1 | 0.21 ± 0.04 | 0.32 ± 0.26 | 0.79 ± 0.16 | 140 ± 51 |
| HPMC | 100 | 0.2 | 1 | 0.19 ± 0.04 | 0.56 ± 0.33 | 0.68 ± 0.10 | 117 ± 81 |
| HPMC | 100 | 0.3 | 1 | 0.64 ± 0.91 | 1.79 ± 1.08 | 0.49 ± 0.11 | 2 ± 3 |
| Kollidon VA64/MCC | 20/80 | 0.1 | 7 | 0.94 ± 0.59 | 10.73 ± 8.90 | 0.25 ± 0.20 | 34 ± 13 |
| Kollidon VA64/MCC | 20/80 | 0.2 | 7 | 2.57 ± 0.25 | 19.77 ± 3.84 | 0.17 ± 0.04 | 26 ± 3 |
| Kollidon VA64/MCC | 10/90 | 0.1 | 7 | 2.23 ± 0.48 | 19.01 ± 6.39 | 0.14 ± 0.04 | 20 ± 7 |
| Kollidon VA64/MCC | 10/90 | 0.2 | 7 | 7.68 ± 0.66 | 15.95 ± 2.92 | 0.45 ± 0.04 | 8 ± 1 |
| Kollidon VA64/MCC | 10/90 | 0.3 | 7 | 17.71 ± 4.91 | 102.64 ± 20.85 | 0.26 ± 0.04 | 8 ± 1 |
| Kollidon VA64/MCC | 40/60 | 0.1 | 7 | 1.56 ± 0.36 | 4.33 ± 1.20 | 0.26 ± 0.04 | 30 ± 2 |
| Kollidon VA64/MCC | 40/60 | 0.2 | 7 | 3.54 ± 1.86 | 12.77 ± 5.10 | 0.18 ± 0.07 | 22 ± 9 |
| Kollidon VA64/MCC | 40/60 | 0.3 | 7 | 4.64 ± 0.57 | 26.91 ± 0.69 | 0.17 ± 0.01 | 19 ± 2 |
| Kollidon VA64/Mannitol | 10/90 | 0.1 | 7 | 3.62 ± 0.34 | 7.22 ± 6.26 | 0.42 ± 0.14 | 12 ± 3 |
| Kollidon VA64/Mannitol | 10/90 | 0.2 | 7 | 3.23 ± 0.22 | 9.48 ± 6.05 | 0.52 ± 0.13 | 19 ± 2 |
| Kollidon VA64/Mannitol | 10/90 | 0.3 | 7 | 9.54 ± 3.52 | 163.35 ± 15.07 | 0.11 ± 0.02 | 18 ± 5 |
| Kollidon VA64/MCC/NaCMCXL/MgSt | 20/77.5/2/0.5 | 0.3 | 7 | 61.63 ± 42.58 | 138.40 ± 104.64 | 0.28 ± 0.11 | 7 ± 9 |
| Kollidon VA64/MCC/NaCMCXL/MgSt | 20/76/2/2 | 0.3 | 7 | 6.80 ± 1.72 | 125.28 ± 26.18 | 0.20 ± 0.07 | 9 ± 1 |
| Kollidon VA64/MCC/NaCMCXL/SSF | 20/77.5/2/0.5 | 0.3 | 7 | 9.45 ± 2.92 | 205.30 ± 116.84 | 0.20 ± 0.07 | 12 ± 6 |
| Kollidon VA64/MCC/NaCMCXL/SSF | 20/76/2/2 | 0.3 | 7 | 7.08 ± 1.20 | 90.86 ± 18.45 | 0.29 ± 0.04 | 9 ± 0 |
| Kollidon VA64(medium)/MCC/NaCMCXL | 20/78/2 | 0.3 | 7 | 15.72 ± 5.60 | 184.64 ± 170.76 | 0.26 ± 0.13 | 7 ± 2 |
| Kollidon VA64(fine)/MCC/NaCMCXL | 20/78/2 | 0.3 | 7 | 6.85 ± 1.24 | 21.31 ± 5.77 | 0.44 ± 0.05 | 9 ± 1 |

Supplement: Characteristic parameters (average and standard deviation) determined by fitting of water uptake and swelling data of presented experiments according to the empirical model using Equations 2, 3 and 4: kinetic constant for pore penetration k_P_, kinetic constant for swelling k_S_, swelling exponent n and transition point t*. The standard deviation includes any deviation, including tablet manufacturing variations, measurement errors from water uptake and swelling analysis, and deviations from the mathematical fitting procedure.
